# On the Shape of the Stethoscope

**Published:** 1855-08

**Authors:** 


On the Shape of the Stethoscope. By Professor Fick.—

Professor Fick, in order to avoid a fine humming noise which he finds that the application of the ordinary stethoscope to the ear invariably produces, recommends the employment of a tube without an ear-piece, which he introduces into the meatus. He has used a stethoscope of this construction for many years, and states that it presents the thoracic noises more distinctly, and possesses the advantage of allowing the head of the observer to be more extensively moved and more easily applied in various postures than the ordinary stethoscope. He explains the adventitious murmur by the compression the ordinary instrument exerts upon the tragus.—*Med. Chir. Rev.*

